# New Therapeutic Opportunities for the Treatment of Squamous Cell Carcinomas: A Focus on Novel Driver Kinases

**DOI:** 10.3390/ijms22062831

**Published:** 2021-03-11

**Authors:** Ryan Bensen, John Brognard

**Affiliations:** Laboratory of Cell and Developmental Signaling, Center for Cancer Research, National Cancer Institute, Frederick, MD 21702, USA; bensenrc@nih.gov

**Keywords:** oncogenes, kinases, cell signaling, PI3K, squamous carcinomas, receptor tyrosine kinases, EGFR, PKC, MAPK, 3q amplicon

## Abstract

Squamous cell carcinomas of the lung, head and neck, esophagus, and cervix account for more than two million cases of cancer per year worldwide with very few targetable therapies available and minimal clinical improvement in the past three decades. Although these carcinomas are differentiated anatomically, their genetic landscape shares numerous common genetic alterations. Amplification of the third chromosome’s distal portion (3q) is a distinguishing genetic alteration in most of these carcinomas and leads to copy-number gain and amplification of numerous oncogenic proteins. This area of the chromosome harbors known oncogenes involved in squamous cell fate decisions and differentiation, including *TP63*, *SOX2*, *ECT2*, and *PIK3CA*. Furthermore, novel targetable oncogenic kinases within this amplicon include *PRKCI*, *PAK2*, *MAP3K13*, and *TNIK*. TCGA analysis of these genes identified amplification in more than 20% of clinical squamous cell carcinoma samples, correlating with a significant decrease in overall patient survival. Alteration of these genes frequently co-occurs and is dependent on 3q-chromosome amplification. The dependency of cancer cells on these amplified kinases provides a route toward personalized medicine in squamous cell carcinoma patients through development of small-molecules targeting these kinases.

## 1. Introduction

**Figure 1:**
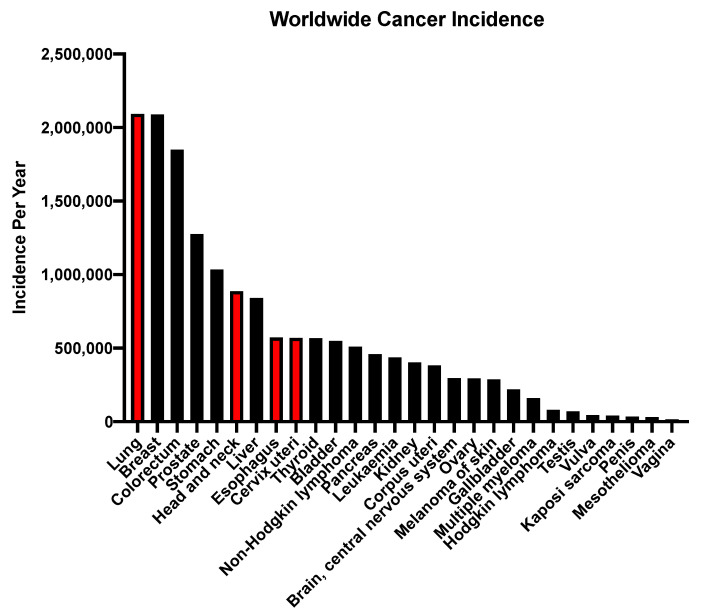
Worldwide incidence of the major types of human cancers. Red bars indicate cancers that have a squamous cell carcinoma (SCC) subtype. Data acquired from GLOBOCAN 2018.

Squamous cell carcinomas (SCCs) constitute a large proportion of all human cancers with little to no targetable therapies available (Figure 1). Outside of SCC of the skin, more than two million new cases of SCCs are diagnosed each year worldwide, primarily located in the lung (LSCC), head and neck (HNSCC), esophagus (ESCC), and the cervix (CvSCC), accounting for more than 1.5 million deaths per year [1,2,3,4,5]. Approximately 25% of lung [2], >90% of head and neck [3], 90% of esophageal [4], and >90% of cervical [5] cancers are classified as SCCs. Despite our increased knowledge and understanding of these cancers, there have been minimal improvements in medical treatments in the past three decades.

The diagnosis and treatment of SCCs typically follows the anatomical tissue distribution of the cancer; for example, LSCC would be treated differently than HNSCC. However, SCCs are closely linked genetically, with mutations in cell-cycle proteins and increased expression of oncogenic molecules involved in squamous cell fate and/or differentiation pathways [6]. Furthermore, the human papillomavirus (HPV) is a causative agent in nearly all cases of CvSCC and ~25% of HNSCCs, exhibiting a distinct genetic signature and unifying clinical outlook [7]. Within HPV-negative (HPV-) SCCs, the most prominent mutation is in TP53 (both loss and a high frequency of gain-of-function mutations are observed), resulting in deregulation of many genes involved in cell survival, invasion, and proliferation [6]. A deep deletion of the tumor suppressor, *CDKN2A* (p16)*,* is a prominent genetic alteration in HPV- SCCs. The E6 and E7 HPV viral proteins inhibit the p53 and p105-RB proteins (binding partners of p16), respectively, in HPV+ tumors, thereby making genetic mutations of *TP53* and *CDKN2A* uncommon in these carcinomas [6,8].

Within both HPV+/− SCC tumors, a unifying genetic alteration in >75% of clinical samples is copy-number gain (CNG) (GISTIC score of +1) or amplification (GISTIC score of +2) of chromosome 3q 26-29 (3q amplicon), harboring key oncogenic drivers involved in SCC cell fate determination, including *TP63*, *SOX2, ECT2,* and *PIK3CA* [9]. The 3q amplicon includes ~200 protein coding genes, of which several have been identified as potential oncogenic drivers in SCCs with a high degree of cooperativity [9,10]. Although there has been an increase in research into these oncogenes, there have been no U.S. Food and Drug Administration (FDA)-approved therapeutics targeting any of these proteins in SCCs to date [9,10].

In this review, we will summarize the discovery of new druggable proteins, focusing on kinases within the 3q amplicon, and their application in the development of novel SCC treatments. We will highlight the current approaches taken toward treating these SCCs along with the experimental clinical trials on kinases being conducted. We review resident genes of the 3q amplicon including the oncogenic kinases *PIK3CA* and *PRKCI*, while providing evidence for novel druggable kinases including *MAP3K13*, *TNIK*, and *PAK*2. This review is intended to provide a better understanding of SCCs and highlight personalized treatment options targeting druggable kinases in SCCs.

## 2. Etiology

### 2.1. Lung Squamous Cell Carcinoma

LSCC is one of the most predominant forms of SCC, accounting for ~500,000 new cases per year [2]. Although still common, its incidence has dropped over the years due to a decrease in tobacco smoking, which is the primary causative agent of LSCC [2]. Unlike lung adenocarcinoma, the majority of patients diagnosed with LSCC are former or current heavy smokers. Tobacco carcinogenesis produced by smoking results in LSCC being characterized by a high somatic mutational burden. Although this cancer is linked to genetic instability, the DNA mutations targeted for adenocarcinoma therapy, including EGFR mutations and echinoderm microtubule-associated protein-like 4 (EML4)-anaplastic lymphoma kinase (ALK) translocations, are essentially absent in LSCC [2,11]. Therefore, the standard of care for metastatic LSCC is chemotherapy, traditionally using a dual platinum/taxane-compound approach, such as carboplatin/paclitaxel [2].

There have been some minor steps toward the development of targeted therapies for LSCC (Table 1). The first FDA-approved compound outside of traditional chemotherapy for LSCC is the antiangiogenetic inhibitor ramucirumab, approved in 2014, which is only used in the second-line setting. A more personalized, targeted therapeutic, necitumumab, gained FDA approval in 2015. Necitumumab is a monoclonal antibody inhibitor of the epidermal growth factor receptor (EGFR) specifically approved as a first-line treatment for LSCC patients combined with chemotherapy treatment. Similarly, afatinib gained FDA approval in 2016 as an EGFR/HER2 inhibitor, although it is administered only in a second-line setting for LSCC patients whose disease has progressed following platinum chemotherapy. The only other FDA-approved therapeutics for LSCC are the immune checkpoint inhibitors, nivolumab (2015), pembrolizumab (2015), atezolizumab (2016), and durvalumab (2018). Although there have been numerous developments for the treatment of LSCC, a recent analysis of these treatments in the lung cancer master protocol (Lung-MAP) has shown that only 7% and 17% of patients have had a positive response to targeted therapies or immune checkpoint inhibitors, respectively, highlighting the critical need for new druggable targets in LSCC [12].

### 2.2. Head and Neck Squamous Cell Carcinoma

HNSCC is also a predominant form of SCC with >600,000 new cases each year [3,13]. Much like LSCC, tobacco use is a causative agent of HNSCC; however, alcohol consumption and HPV, particularly the HPV-16 genotype, are additional major risk factors [3]. HNSCC arises from the mucosal surfaces of the nasal and oral cavity, along with the oropharynx, larynx, and hypopharynx. Much like LSCC, the standard of care for metastatic HNSCC has long been a taxane/platinum doublet, platinum/5-fluorouracil (5FU) doublet, or, more recently, a taxane/cisplatin/5FU (TPF) treatment [3]. However, the discovery of the overexpression and dependency on EGFR in HNSCC led to the development of the EGFR monoclonal antibody, cetuximab, which gained FDA approval in 2006 as a first-line agent in combination with radiation or as a single, second-line agent for non-metastatic HNSCC. In 2011, cetuximab was granted FDA approval as a first-line treatment in combination with chemotherapy for metastatic HNSCC. EGFR is overexpressed in ~90% of HNSCC patients and drives the cancer through downstream MAPK and phosphatidylinostiol-3-kinase (PI3K)/AKT/mammalian target of rapamycin (mTOR) signaling [14]. Cetuximab is the only FDA-approved targeted therapy for HNSCC outside of immune checkpoint inhibitors and together with a platinum/taxane or 5FU doublet forms the EXTREME regimen as the first-line treatment for metastatic HNSCC.

### 2.3. Esophageal Squamous Cell Carcinoma

ESCC is a highly fatal malignancy, accounting for ~500,000 cases each year with a five-year survival rate ranging from 15 to 20% [15]. The etiology of ESCC is multifactorial, dependent on population, gender, tobacco use, alcohol use, and diet [4,15]. ESCC has the highest incidence in the eastern world, particularly eastern-to-central Asia and the south-western portion of Africa; however, esophageal adenocarcinoma (unique from ESCCs) is becoming more prevalent in western countries along with parts of Europe and Australia [4]. The standard of care for ESCC typically involves a multimodal treatment, incorporating either local radiofrequency ablation or endoscopic treatment followed by a platinum/taxane doublet chemotherapy. There have yet to be any targeted, personalized therapies for ESCC, with the only non-chemotherapy, FDA-approved drugs being the immune checkpoint inhibitors, pembrolizumab and nivolumab, which were approved in 2019 and 2020, respectively.

### 2.4. Cervical Squamous Cell Carcinoma

CvSCC accounts for ~500,000 cases each year, predominantly in underdeveloped and developing countries (85% of deaths) [5]. The highest incidences occur in Central and South America, southern Asia, the Caribbean, and Sub-Saharan Africa. The majority of CvSCC cases arise from HPV infection, detected in ~95% of patients with CvSCC [5,16]. The development of prophylactic vaccinations against HPV in the early 2000s has provided primary prevention, mainly in the developed world [17]. Furthermore, secondary screening approaches using cytologic cell examination on a Papanicolaou smear or DNA testing of HPV have largely prevented this cancer in the developed world, but it continues to be a global health concern in less-developed countries [5]. The standard of care for advanced CvSCC is adjuvant chemotherapy with cisplatin. The antiangiogenic compound, bevacizumab, gained FDA approval in 2014, and the immune checkpoint inhibitor, pembrolizumab, gained FDA approval in 2018 as additional options for treating advanced CvSCC. There are currently no other FDA-approved targeted therapeutics for CvSCC.

## 3. Kinases as Targets for Experimental Therapeutics

### 3.1. Next-Generation Sequencing

The search for novel, antineoplastic therapeutic targets has been heavily aided by next-generation sequencing (NGS) advances. Projects such as The Cancer Genome Atlas (TCGA) have provided a wealth of genetic data for multiple types of cancers. Gene mutations that result in chronic proliferation, known as Mut-driver genes, have been identified in only 125 of the 20,000 protein-coding genes [18]. All the clinically approved drugs that target these Mut-driver genes are directed against kinases, largely due to our knowledge of their physiology and the relative simplicity of inhibiting catalytic, enzymatic activity [18]. A hallmark of cancer is dysregulation of cell signaling pathways through alteration in kinase expression and/or catalytic activity mediated by genetic mutations, translocations, gene amplifications, or deletions of regulatory domains. Historically, much of our clinical success in treating cancers has come from developing molecules targeting these oncogenic kinases. Bioinformatic screens have suggested that several understudied kinases remain to be targeted for personalized therapy [19,20]. There are currently numerous molecules undergoing clinical trials that target both well-characterized kinases and some understudied kinases, which are further described below (Table 2).

### 3.2. EGFR

The EGFR (HER1) inhibitors, cetuximab, afatinib, and necitumumab, remain the only FDA-approved, first-line, targeted therapeutic for SCC to date. EGFR belongs to the ErbB family of receptor tyrosine kinases which comprises EGFR, HER2, HER3, and HER4. Many solid tumors have an upregulation of EGFR, including most LSCCs (>80%) [21] and HNSCCs (>90%) [22]. Multiple cellular mechanisms can lead to the upregulation or activation of EGFR, including gene amplification and/or protein overexpression, genetic mutations to the extracellular domain or kinase domain, or exon 19 truncations [23]. These mutations and/or overexpression lead to activation of oncogenic downstream signaling cascades, primarily the MAPK and PI3K-mTOR pathways (reviewed in [21,24,25]). The dependency on EGFR signaling in numerous cancers has led to the development of multiple inhibitors belonging to two different families: monoclonal antibodies, which block the extracellular domain, or tyrosine kinase inhibitors (TKIs), which bind the kinase domain [24]. FDA-approved monoclonal antibodies targeting EGFR include the aforementioned cetuximab (head-and-neck/colorectal cancers), necitumumab (non-small-cell lung cancer (NSCLC)), and panitumumab (colorectal). FDA-approved TKIs include erlotinib (NSCLC/pancreatic cancer), gefitinib (NSCLC), afatinib (NSCLC), osimertinib (NSCLC), dacomitinib (NSCLC), and lapatinib (breast). Additionally, the EGFR/HER2-targeting TKI poziotinib is currently under Phase 2 clinical trials for patients harboring an Exon 20 insertion with HNSCC (Table 2). Although our knowledge of this receptor has increased dramatically over the past few decades, patients typically acquire resistance to EGFR inhibitors, either through mutations in EGFR itself or mutations in components of the downstream signaling pathways, including MAPK, PI3K, and mTOR, resulting in the need to identify other drivers in SCCs.

### 3.3. PI3K

Class 1 PI3Ks are the main class associated with cancer responsible for the phosphorylation of phosphatidylinositol 4,5-bisphosphate (PtdIns-4,5-P_2_) producing phosphatidylinositol 3,4,5-trisphosphate (PtdIns 3,4,5-P_3_). Class 1 consists of four different catalytic isoforms, p110α, β, γ, and δ, encoded by *PIK3CA*, *PIK3CB*, *PIK3CG*, and *PIK3CD* genes, respectively. PtdIns 3,4,5-P_3_ is a second-messenger lipid generated by PI3K, which activates several downstream signaling cascades. Effector proteins of PtdIns 3,4,5-P_3_ all contain a pleckstrin homology (PH) domain capable of binding PtdIns 3,4,5-P_3_. These effector proteins include, but are not limited to, AKT, phosphoinositide-dependent-kinase-1 (PDK1), Bruton’s tyrosine kinase, and guanine nucleotide-exchange factors (reviewed in [26,27,28,29]). AKT activation is the most canonical downstream effector of PtdIns 3,4,5-P_3_ being nearly universally activated upon PtdIns 3,4,5-P_3_ production. AKT is recruited to the plasma membrane along with its upstream kinase, PDK1, which phosphorylates AKT at Thr 308. mTOR complex 2 (mTORC2) comprises the protein subunits rapamycin-insensitive companion of mTOR (RICTOR), mammalian stress-activated protein kinase interaction protein 1 (mSIN1), mammalian lethal with sec-13 protein 8 (mLST8), and protein observed with RICTOR 1 and 2 (Protor1/2) as well as phosphorylates AKT at S473 leading to the complete activation of AKT and results in an increase in cellular proliferation and survival. The tumor suppressor protein, PTEN, dephosphorylates PtdIns 3,4,5-P_3_ at the 3′ position yielding PtdIns-4,5-P_2_, thereby terminating the activation of downstream AKT signaling.

Activation of PI3K contributes to tumorigenesis and resistance to numerous cancer therapies, including EGFR inhibition [30,31]. The PI3K pathway is dysregulated in nearly all human cancers and inhibition leads to decreased proliferation and cell death. This essentiality has led to the development of multiple kinase inhibitors of PI3K consisting of three generations. Generation one includes pan-PI3K inhibitors, largely used in preclinical models to study this complex pathway with little success in the clinic, mainly due to poor pharmacodynamic properties [26,27]. The second generation of compounds exhibit better pharmacodynamic properties and include isoform selective PI3K inhibitors [27]. Lastly, the third generation of compounds are dual PI3K/mTOR inhibitors, proposed to theoretically have the highest antineoplastic activity [32].

Currently, there are no FDA-approved PI3K inhibitors for any SCC, but there are multiple compounds in clinical trials (Table 2). Copanlisib is an FDA-approved PI3Kα -isoform-specific inhibitor for patients with relapsed follicular lymphoma and is currently under clinical trials for HNSCC and NSCLC. Similarly, Alpelisib is also an FDA-approved PI3Kα -selective inhibitor currently under clinical trials for HNSCC. The PI3Kδ/γ selective inhibitor Duvelisib is an FDA-approved drug for lymphocytic leukemia/small lymphocytic lymphoma and follicular lymphoma and is currently under clinical trials for HNSCC. Lastly, the PI3K/mTOR targeting compound Bimirlisib and the pan-PI3K inhibitor Buparlisib are also under clinical trials for HNSCC.

### 3.4. JAK

Janus kinase (JAK) is a nonreceptor tyrosine kinase that interacts with type I and type II cytokine receptors to activate the JAK-STAT pathway involved in cell proliferation and death [33]. There are four members of the JAK family: JAK1, JAK2, JAK3, and TYK2, with JAK2 having a more prominent role in cancer. Aside from transcription factor activation of STAT proteins, JAKs can also function upstream of MAPK and PI3K/AKT signaling pathways through phosphorylation of tyrosine kinase receptors, leading to the activation of the MAPK and PI3K signaling cascades [34]. This crosstalk provides another route toward drug resistance and further complicates the treatment of SCCs. The FDA-approved JAK1/2 inhibitor for myelofibrosis, ruxolitinib, is currently under clinical trials for HNSCC.

### 3.5. ATR

Ataxia telangiectasia and Rad3-related protein (ATR) is a serine/threonine protein kinase belonging to the phosphatidylinositol 3-kinase-related kinase (PIKK) family which includes five other members: ataxia telangiectasia mutated (ATM), DNA-dependent protein kinases (DNA-PKcs), mTOR, transformation/transcription domain-associated protein (TRRAP), and SMG1. ATR, along with ATM, are large sensory proteins that respond to DNA damage and regulate multiple essential cellular functions, including cell-cycle checkpoint activation and DNA damage repair. ATM is activated upon the detection of double-stranded DNA breaks, and loss of ATM protein expression is a common occurrence in cancer cells [35]. ATR recognizes single-stranded DNA damage that accumulates when DNA is being repaired through mechanisms including nucleotide excision repair and homologous recombination and is essential for the viability of replicating cells. ATR and ATM have a variety of substrates but uniquely have a strong preference to phosphorylate Ser/Thr residues that are followed by Gln [36]. The best-studied substrate of ATR is the checkpoint kinase 1 (CHK1) through phosphorylation at Ser317 and Ser345. Phosphorylation of CHK1 by ATR induces phosphorylation and deactivation of CDC25 phosphatases that are responsible for the activation of cyclin-dependent kinases (CDKs), resulting in restricted mitotic entry [36].

Loss of ATR activity allows cells to avoid activation of cell-cycle checkpoints and continue with proliferation in the presence of DNA damage generated by chemotherapeutics. ATR inhibitors prevent the ATR-dependent DNA repair mechanisms induced by cisplatin chemotherapy, for example, and this potentiates the effect of the chemotherapy and results in mitotic catastrophe and cellular death [37]. Currently, the ATR inhibitor ceralasertib is under clinical trials for HNSCC.

### 3.6. VEGFR

Vascular endothelial growth factor receptor (VEGFR) is a well-studied receptor tyrosine kinase (RTK) with a biological function to regulate vasculogenesis and angiogenesis. There are three members of the VEGFR family: kinase insert domain-containing receptor (KDR/VEGFR-2), Fms-like tyrosine kinase (FLT1/VEGFR-1), and FLT4 (VEGFR-3). VEGFR-2 is the primary kinase receptor of VEGF-A for activation of angiogenesis, cellular proliferation, and survival. VEGFR-2 expression is greatly increased within the majority of solid tumors and serves as a vital signal transducer for cellular growth and survival, making it an intriguing pharmacological target. VEGF-A binding to VEGFR-2 activates a downstream proangiogenic signaling cascade, primarily through a PLCγ-PKC-MAPK axis [38]. Numerous inhibitors have been developed against VEGFRs, with ramucirumab being the only FDA-approved inhibitor for SCC targeting VEGFR-2 (Table 1). However, bevacizumab is an FDA-approved inhibitor of free VEGF-A for CvSCC. There are multiple compounds currently in clinical trials evaluating the efficacy of anti-VEGFR activity for SCC therapy. These compounds can be either specific for VEGFR-2 or can be pan-kinase inhibitors that target multiple kinases including the VEGFRs (Table 2).

### 3.7. FGFR

Fibroblast growth factor receptors (FGFRs) comprise a family of four highly conserved receptor tyrosine kinases (FGFR1–4) and an additional receptor, FGFR5, which lacks an intracellular kinase domain. Receptor activation through the binding of fibroblast growth factor (FGF) activates a downstream signaling cascade mediated primarily through FGFR substrate 2 (FRS2) and PLCγ, resulting in cellular survival and proliferation through MAPK and PI3K/AKT signaling [39]. Other pathways, including the STAT pathway, can also be activated through FGF binding [39]. FGFR1 amplification is present in approximately 10–30% of SCCs and represents a bona fide candidate for targeted therapy [40]. Currently, the pan-FGFR inhibitor rogaratinib is in phase 2 clinical trials for pretreated LSCC patients (Table 2).

## 4. q Amplicon Kinase Targets

### 4.1. q Amplicon

Gains or amplifications of the distal region of the 3q chromosome, particularly 3q26-29 (3q amplicon), is a common genetic phenotype present in more than 75% of patients with LSCC, HNSCC, ESCC, and CvSCC, while also being prevalent in ovarian and uterine carcinomas [9]. Many research groups set out to identify oncogenic drivers within this region, with limited success [8,9,10,41]. SOX2 and TP63 are two of the most well characterized oncogenes within the 3q amplicon. They are selectively overexpressed in the majority of SCCs responsible for the regulation of squamous cell survival and differentiation [9]. TP63 is a direct transcriptional target of SOX2 and controls numerous genes involved in cellular differentiation and growth, including the aforementioned FGFR1 [9,42]. However, cellular reliance on p63 and SOX2 seems to diminish as the tumor grows, and increasing evidence suggests the loss of these proteins is correlated with tumor metastasis [43,44]. No FDA-approved small molecule inhibitors exist for either SOX2 or p63, so other targetable oncogenic proteins within the 3q amplicon need to be identified for the development of efficacious SCC therapies.

**Figure 2:**
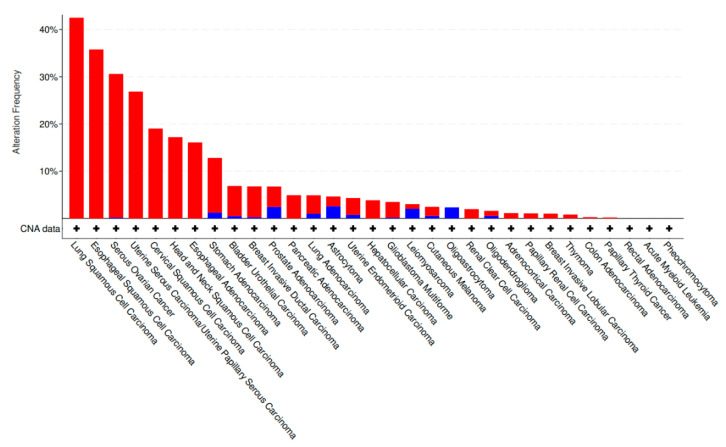
Amplification (red)/deletion (blue) frequency of the 3q amplicon kinase genes *PIK3CA*, *PRKCI*, *MAP3K13, TNIK*, and *PAK2* in a The Cancer Genome Atlas (TCGA) pan-cancer analysis using cBioPortal. A minimum cutoff of 85 clinical samples was used per cancer type.

**Figure 3:**
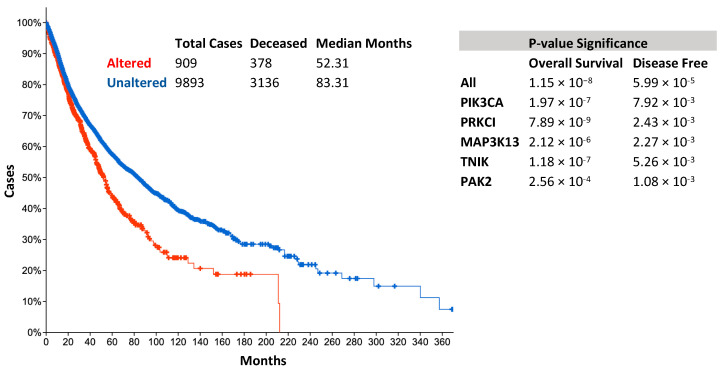
Survival statistics and amplification of 3q amplicon genes.

A search of TCGA data sets using cBioPortal for kinases within the 3q amplicon has identified several potential oncogenes for targetable therapy, including *PIK3CA*, in addition to *PRKCI*, *MAP3K13*, *TNIK*, and *PAK2*. There is preferential amplification of these genes with at least one gene being amplified in more than 40% of LSCC, 35% of ESCC, 19% of CvSCC, and 15% of HNSCC compared to other cancers (Figure 2). Ovarian and uterine carcinoma also have a high frequency of the 3q amplification (greater than 25% of all cases) (Figure 2). Amplification of these genes, either individually or together, correlates with a significantly lower survival outcome in a pan-cancer analysis (Figure 3), supporting the notion that these kinases are important for cancer development and are linked to increased mortality.

### 4.2. PRKCI

Protein Kinase C iota (PKCι) is a member of the atypical protein kinase C isozymes coded by the *PRKCI* gene located on chromosome 3q26. PKCι functions differently than traditional PKCs in that the catalytic activity of PKCι is not regulated by calcium, diacylglycerol, or phosphatidylserine [45,46,47]. Rather, the diverse functions of PKCι are proposed to be regulated through protein–protein interactions mediated through its Phox Bem 1 (PB1) domain [48]. PKCι expression is directly correlated with carcinogenesis through different mechanisms and linked with a poor clinical prognosis [49,50,51,52]. Within LSCC, along with ovarian and pancreatic cancers, the Fields lab has performed pioneering studies to demonstrate that PKCι regulates proliferation and invasion through a PKCι-PAR6-ECT2-RAC1-PAK-MEK-ERK signaling pathway [53,54,55]. Furthermore, in LSCC, the Fields lab has shown that PKCι is co-expressed with SOX2 and directly phosphorylates SOX2, thereby recruiting it to the promotor of hedgehog acyltransferase, which results in a stem-like phenotype [56]. In addition, PKCι expression correlated significantly with lymph node metastasis, tumor size, and clinical stage in ESCC through a PKCι-SKP2-PI3K/AKT-dependent pathway [57,58].

**Figure 4:**
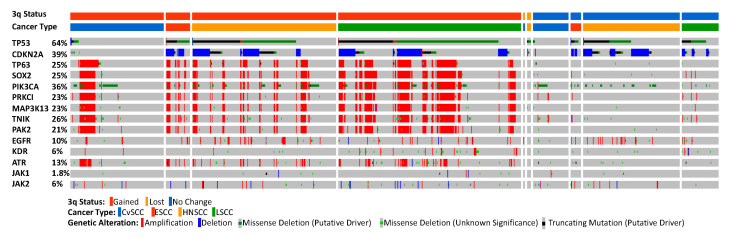
Alteration and mutation frequency of multiple genes within cervix (CvSCC), esophagus (ESCC), lung (LSCC), and head and neck (HNSCC) TCGA data sets using cBioPortal. Genes include the most altered gene, *TP53*; the HPV biomarker, *CDKN2A*; the 3q amplicon genes; and current genes being targeted in clinical trials. A total of 1317 patients were analyzed for gene alteration/mutation, of which 957 were analyzed for 3q status.

TCGA analysis of PRKCI shows CNGs within LSCC, HNSCC, CvSCC, and ESCC being amplified in 23% of all patients evaluated. PRKCI was also observed to be significantly coamplified with SOX2 and other 3q amplicon genes (*p*-value < 0.001, LOG2 > 3) (Figure 4). Small-molecule binding to PKCι’s PB1 domain, rather than traditional ATP competitive inhibition, is an exciting route toward inhibiting the vast functions of PKCι. The gold compound aurothiomalate specifically inhibits PB1–PB1 domain binding of PKCι and PAR6 through specificity for a cysteine residue unique to PKCι and was safely evaluated in a clinical setting [59]. Furthermore, the non-kinase oncogenic functions of PKCι enable opportunities for small-molecule PROTAC (proteolysis targeting chimera) development, potentially eliciting further therapeutic efficacy unachievable through conventional ATP-competitive kinase inhibitors. The diverse oncogenic functions of PKCι mediated through PB1 protein interactions in multiple different cancers that will be activated through CNGs of the 3q amplicon make PKCι an exciting therapeutic target for small-molecule inhibitors and PROTAC development.

### 4.3. MAP3K13

The leucine zipper-bearing kinase (LZK) encoded by the MAP3K13 gene is an understudied protein kinase belonging to the mixed lineage kinase (MLK) family. LZK was first identified as a regulator of the c-Jun N-terminal kinase (JNK) pathway through phosphorylation of MAP2Ks (MKK4 and MKK7) [60,61]. LZK was also shown to regulate NF-kB activity by activating IKK and binding to antioxidant protein 1 (AOP-1) [62]. More recently, LZK was demonstrated to play a synergistic role with its closest homologue, dual leucine zipper kinase (DLK), as a positive regulator of axon growth through the LZK-MKK4-JNK pathway [63]. Inhibitors of both LZK and DLK have been shown to protect against neuron degradation in vitro and have therapeutic activity in Parkinson’s disease mouse models [64].

Our group recently demonstrated that LZK is essential for the survival of HNSCC cell lines harboring the 3q amplicon. LZK mediates its oncogenic function independent of JNK and NF-κB signaling and utilizes a novel mechanism to stabilize mutant gain of function (GOF) p53, thereby regulating AMP-activated protein kinase (AMPK) and p21 expression. [65] Analysis using the TCGA data set including LSCC, HNSCC, CvSCC, and ESCC shows that MAP3K13 is amplified in 23% of clinical samples (Figure 4). Further research is needed to evaluate LZK’s potential oncogenic function and mechanism of action in 3q-amplicon-positive LSCC, ESCC, and CvSCC. CvSCC would be particularly interesting because HPV is a prevalent causative agent and regulates the degradation of p53 in CvSCC, contradictory to LZK’s oncogenic function of stabilizing GOF mutant p53 in HNSCC.

### 4.4. TNIK

Traf2-and NCK-interacting kinase (TNIK) was first identified as cytoskeletal regulating protein that activates the JNK pathway [66]. It was further identified as a colorectal oncogene that activates Wnt signaling by directly phosphorylating T-cell transcription factor 4 (TCF4) and binding to β-catenin [67,68]. Within gastric cancer, however, TNIK has a Wnt-independent oncogenic function through AKT activation and cell autophagy [69]. TNIK is also an essential gene in tumors harboring the 3q amplicon, but an in-depth evaluation into the function of the protein, primarily in SCCs, has been lacking. Recently, however, our group has identified TNIK as a therapeutic target in LSCC. Pharmacological inhibition or genetic knockdown of TNIK resulted in reduced growth in vitro and in vivo through a mechanism whereby TNIK mediates phosphorylation of Merlin, which is necessary for the activation of focal adhesion kinase (FAK) [70].

TCGA analysis shows TNIK has an important function in SCCs and correlates with a significant decrease in patient survival when there are amplifications of the gene (Figure 3). Amplification of TNIK is identified in 26% of LSCC, HNSCC, ESCC, and CvSCC patients and is significantly co-expressed with other members of the 3q amplicon (Figure 4). TNIK is a promising, novel therapeutic target in SCC tumors harboring the 3q amplicon.

### 4.5. PAK2

p21-activated kinase 2 (PAK2) was initially identified as an RHO GTPase, CDC42, and an RAC1 binding partner [71]. PAK2 is a multifunctional protein kinase involved in cytoskeletal and chromatin remodeling, proliferation, and apoptosis [72,73]. PAK2 is unique in that it has both pro- and anti-apoptotic functions. Its apoptotic function is activated upon caspase proteolytic cleavage, resulting in the formation of PAK2p34. The formation of PAK2p34 correlates with cellular death, which is activated by multiple stimuli and cellular stressors [72,74]. Furthermore, PAK2 functions as a negative regulator of MYC by directly phosphorylating MYC and reducing its binding to DNA either through inhibiting dimerization with Max (S373/T400) or direct inhibition of DNA binding (T358) [75]. Conversely, PAK2 is also reported to upregulate the expression of MYC in a β-catenin-dependent manner that induces PKM2 expression, leading to cellular proliferation in an HNSCC model [76]. Additionally, full-length PAK2 functions as a proliferative oncoprotein by preventing tumor necrosis factor alpha (TNFa)-induced cellular death through phosphorylation of BAD and regulation of ERK, JNK, and p38 pathways [77]. Lastly, PAK2 induces the phosphorylation of the Nf2 tumor suppressor gene product, Merlin, in a RAC/CDC42-dependent manner [78]. Thus, PAK2 has numerous complex regulatory functions driven by unique genetic backgrounds to either promote or suppress tumorigenesis.

Analysis of PAK2 utilizing cBioPortal within LSCC, HNSCC, ESCC, and CvSCC shows amplification of PAK2 in 21% of the clinical samples (Figure 4). Amplifications of PAK2 correlated with significantly lower patient survival (Figure 3). PAK2′s diverse cellular functions require an in-depth analysis in 3q-amplicon-positive SCCs and make PAK2 a promising targetable kinase for antiproliferative small molecules.

## 5. Concluding Remarks

SCCs of the lung, head and neck, esophagus, and cervix continue to be global health concerns with little development in targeted therapies over the last three decades. Current steps toward developing therapies for SCCs largely involve either immunotherapy (including immune checkpoint inhibitors recently granted FDA approval) or kinase inhibitors. Promising clinical trials are being conducted on kinase inhibitors that target the squamous cell growth and differentiation pathways, including upstream receptor tyrosine kinases (EGFR, FGFR, and VEGFR) and PI3K (Table 1). However, further oncogenic, druggable protein targets need to be identified within SCCs.

A unifying genetic alteration in all SCCs is the distal amplification of the 3q chromosome resulting in amplifications of key genes involved in squamous cell fate decisions and differentiation, including *TP63*, *SOX2*, *ECT2*, and *PIK3CA*. Numerous research groups have identified other potential oncogenes in this region, including FXR1, CLAPM1, EIF4G, SEC62, and SKIL [79,80,81,82]. More recently, Qian et al. described LSCC’s dependency on FXR1, which formed a complex with and regulated two other 3q amplicon oncogenes, PKCι and ECT2, and was associated with poor clinical outcome when overexpressed [83]. Furthermore, Bochen and Adisurya et al. showed a connection between SEC62 and SOX2 expression affecting lymphatic metastasis and migration in clinical HNSCC samples. Collectively, these studies have identified oncogenic proteins present in the 3q amplicon as strong targets for therapeutic intervention in SCCs; however, there have been no FDA-approved therapeutics targeting these gene products to date, which requires a fresh look into the 3q amplicon for druggable targets.

Our own search of the 3q amplicon has aligned with previous studies and identified the protein products of *PIK3CA, PRKCI*, *MAP3K13*, *TNIK*, and *PAK2* as exciting, plausible therapeutic targets for SCCs. Numerous studies have demonstrated that multiple different cancers both within and outside of SCCs depend on PKCι (recently reviewed in [55]). Furthermore, work from Gupta et al. have shown that PAK2 is essential in HNSCC through regulation of MYC [76], and our own studies have recently elaborated on the oncogenic functions of LZK and TNIK in SCCs through stabilization of mutant p53 and regulation of Merlin, respectively [65,70]. Collectively, these kinases have emerged as druggable oncogenic proteins within SCCs with a high degree of cooperativity and, in addition to established therapies, present a new route toward personalized medicine.

## Figures and Tables

**Table 1:** FDA-approved molecules outside of chemotherapy for treatment of SCCs.

FDA Approved Target Therapies for SCCs		
Head and Neck Cancer	Molecular Target	Year
Cetuximab	EGFR	2006
Pembrolizumab	PD-1	2016
Nivolumab	PD-1	2016
**Non-Small Cell Lung Cancer**		
Afatinib	EGFR	2013
Necitumumab	EGFR	2015
Nivolumab	PD-1	2015
Pembrolizumab	PD-1	2015
Atezolizumab	PD-L1	2016
Durvalumab	PD-L1	2018
Ramucirumab	VEGFR2	2014
**Cervical Cancer**		
Pembrolizumab	PD-1	2018
Bevacizumab	VEGF-A	2014
**Esophageal Cancer**		
Pembrolizumab	PD-1	2019
Nivolumab	PD-1	2020

**Table 2:** Clinical trial compounds targeting kinases in SCCs.

Clinical Trial Kinase Targeting Compounds			
Head and Neck Cancer	Kinase	Phase	NCB
Lenvatinib	VEGFRs	3	NCT04199104
Apatinib	VEGFR2	2	NCT04440917
Axitinib	VEGFRs	2	NCT02762513
Sorafenib	VEGFRs	2	NCT00494182
Ruxolitinib	JAK	2	NCT03153982
Ceralasertib	ATR	1	NCT03022409
Poziotinib	EGFR	2	NCT02216916
Nintedanib	RTK/NRTK	2	NCT03292250
Cabozantinib	c-met/VEGFR	1	NCT03667482
Copanlisib	PI3Kα	1	NCT02822482
Alpelisib	PI3Kα	1	NCT02537223
Duvelisib	PI3Kδ/γ	1	NCT04193293
Abemaciclib	CDK	1	NCT03655444
Palbociclib	CDK	1	NCT03065062
Ribociclib	CDK	1	NCT04000529
Alisertib	AURKA	1	NCT04555837
Ibrutinib	BTK	2	NCT03646461
Bimiralisib	PI3Ks	2	NCT03740100
Buparlisib	PI3Ks	1	NCT02113878
CDX-3379	HER3	2	NCT03254927
**Esophageal Cancer**			
Anlotinib	VEGFRs	2	NCT04063683
Nimotuzumab	EGFR	2	NCT04207918
**Non-Small Cell Lung Cancer**			
Savolitinib	c-met	2	NCT03944772
Rogaratinib	FGFRs	2	NCT03762122
Copanlisib	PI3Kα	1	NCT03735628
Palbociclib	CDK	1	NCT03065062
Ribociclib	CDK	1	NCT04000529
Alisertib	AURKA	1	NCT04479306
Sapanisertib	mTOR	2	NCT02417701

## Data Availability

Cancer incidence data was acquired from the Global Cancer Observatory (GlOBOCAN 2018). All TCGA data was acquired from cBioPortal utilizing the PanCancer Atlas database.
